# Should We Use PPAR Agonists to Reduce Cardiovascular Risk?

**DOI:** 10.1155/2008/891425

**Published:** 2008-01-28

**Authors:** Jennifer G. Robinson

**Affiliations:** Departments of Epidemiology & Medicine, University of Iowa, Iowa City, IA 52242, USA

## Abstract

Trials of peroxisome proliferator-activated receptor (PPAR) agonists have shown mixed results for cardiovascular prevention. Fibrates are PPAR-α agonists that act primarily to improve dyslipidemia. Based on low- and high-density lipoprotein cholesterol (LDL and HDL) effects, gemfibrozil may be of greater cardiovascular benefit than expected, fenofibrate performed about as expected, and bezafibrate performed worse than expected. Increases in both cardiovascular and noncardiovascular serious adverse events have been observed with some fibrates. Thiazolidinediones (TZDs) are PPAR-γ agonists used to improve impaired glucose metabolism but also influence lipids. Pioglitazone reduces atherosclerotic events in diabetic subjects, but has no net cardiovascular benefit due to increased congestive heart failure risk. Rosiglitazone may increase the risk of atherosclerotic events, and has a net harmful effect on the cardiovascular system when congestive heart failure is included. The primary benefit of TZDs appears to be the prevention of diabetic microvascular complications. Dual PPAR-α/γ agonists have had unacceptable adverse effects but more selective agents are in development. PPAR-δ and pan-agonists are also in development. It will be imperative to prove that future PPAR agonists not only prevent atherosclerotic events but also result in a net reduction on total cardiovascular events without significant noncardiovascular adverse effects with long-term use.

## 1. INTRODUCTION

Drugs affecting peroxisome proliferator-activated receptors (PPARs) are of intense interest for regulating disorders of glucose and fatty acid metabolism [1].As an end-stage manifestation of insulin resistance and glucose intolerance, diabetes confers a 2-to-8-fold higher risk of coronary heart disease (CHD), stroke, and mortality [2].Impaired glucose tolerance also contributes to the development of atherogenic dyslipidemia, which is characterized by elevated triglycerides, low high-density lipoprotein (HDL) cholesterol, small dense low-density lipoprotein (LDL) cholesterol, and elevated LDL particle number. Independent of insulin resistance and glucose levels, atherogenic dislipidemia imparts a risk for CHD at least equal to that of the well-characterized risk of isolated, moderate hypercholesterolemia [3].

Agonists of PPAR-*α* and PPAR-*γ* have been evaluated for the long-term prevention of cardiovascular events. Fibrates are low-affinity PPAR-*α* agonists which lower triglycerides by increasing lipolysis and *β*-oxidation of fatty acids [4]. Fibrates also mildly raise HDL and, in some cases, lower LDL. Pharmacologic activation of PPAR-*γ* also lowers triglyceride levels by promoting fatty acid storage [5]. The main benefits of PPAR-*γ* agonists, however, are improvements in glucose homeostasis. Thiazolidinediones (TZDs), or glitazones, are primarily PPAR-*γ* agonists that promote fatty acid oxidation and insulin sensitivity in liver and muscle [1]. These beneficial effects appear to be mediated, at least in part, through inhibition of the release of signaling molecules from adipose tissue that promote insulin resistance, including inflammatory factors such as tumor necrosis factor-*α* (TNF-*α*) and resistin, and stimulating the release of adiponectin. PPAR-*γ* agonism may additionally lower plasma glucose levels via decreased hepatic glucose production. Dual PPAR-*α* and PPAR-*γ* agonists have also been developed. Drugs affecting the more recently identified PPAR-*δ* (also called *β*) are in the early stages of development. PPAR-*δ* is also a powerful regulator of fatty acid catabolism and energy homeostasis and has been shown to prevent weight gain, dyslipidemia, and fatty liver in animals fed high-calorie diets [6, 7]. Given the central role of PPARs in lipid and glucose metabolism, has the promise of PPAR modulation translated into a significant cardiovascular risk reduction benefit from these agents? Several recently completed large trials addressing this question have had mixed results.

## 2. PPAR-*α* AGONISTS: FIBRATES

**Figure 1:**
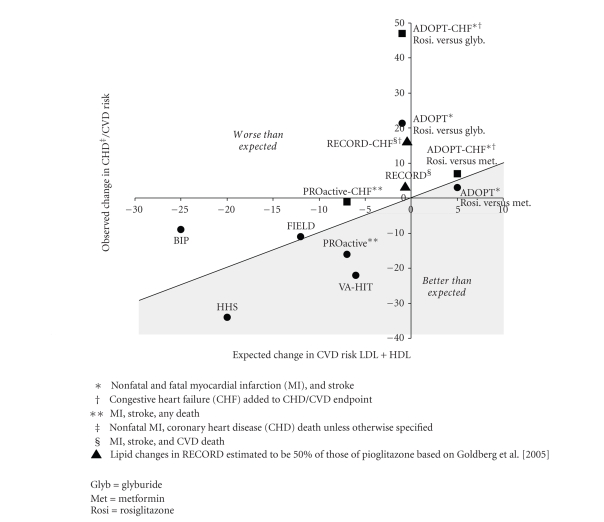
Approximate expected cardiovascular (CVD) risk reduction from percent changes in LDL and HDL versus observed percent reduction in coronary heart disease (CHD) or CVD. Above the slope = 1 line, CVD risk reduction was worse than expected based on lipid changes; below the slope = 1 line, CVD risk reduction was greater than expected based on the lipid changes.

Randomized, placebo-controlled trials have shown that gemfibrozil significantly reduces the risk of CHD in primary and secondary prevention populations of dyslipidemic men, with evidence of a trend toward a decrease in stroke (Table 1) [8, 9]. Less robust results were observed for bezafibrate in subjects with CHD, and for fenofibrate in subjects with diabetes [10, 11]. The cardiovascular benefits of gemfibrozil appear to be greater than expected from changes in LDL and HDL. In the Veterans Affairs HDL Intervention Trial (VA-HIT), a > 20% reduction in CHD and stroke occurred despite no effect on LDL and only a 6% increase in HDL. This reduction in risk was also found to be independent of changes in triglycerides and was largely attributable to the use of gemfibrozil itself [12]. The only other long-term trial with gemfibrozil, the Helsinki Heart Study, also reported a greater reduction in cardiovascular risk than have been expected on the basis of changes in LDL and HDL. Figure 1 is based on the assumption that each 1% decrease in LDL and each 1% increase in HDL are additive and would therefore result in a 2% reduction in cardiovascular risk. Data supporting this assumption comes from clinical trials where each 1% reduction in LDL results in approximately a 1% reduction in the risk of CHD and stroke, regardless of the method by which LDL is lowered [13]. The VA-HIT study found that a 5 mg/dl increase in HDL (16%) reduced risk by 11% [12]. This is consistent with epidemiologic data in which each 1 mg/dl (0.03 mmol, or about a 2-3%, depending on baseline HDL level) increase in HDL is associated with a 2–4% reduction in the risk of CHD events, independent of LDL-C cholesterol levels [14]. It is assumed, but not proven, that raising HDL results in risk reduction additive to that of lowering LDL.

In contrast to the 2 trials with gemfibrozil, the 11% reduction in cardiovascular risk observed in the Fenofibrate Intervention and Event Lowering in Diabetes (FIELD) trial was similar to that expected (about 12%) from average changes in LDL (−9%) and HDL (+3%) between 4 months and the end of the study (Figure 1; Table 2) [11]. The midpoint of the study was chosen due to crossover to statin treatment in both treatment arms. By the end of the trial, 17% of the placebo group and 8% of the fenofibrate group started lipid-lowering therapy, mainly with statins. As a consequence, the lipid parameters for the 2 treatment groups became more similar over time.

The Bezafibrate Infarction Prevention (BIP) study showed a nonsignificant reduction in cardiovascular events of only 9% despite greater changes in LDL and HDL than those observed in FIELD or VA-HIT (Table 2) [8, 10, 11]. Indeed, bezafibrate performed substantially worse than expected from the LDL and HDL changes (Figure 1), suggesting that bezafibrate may have vascular toxicity that counteracts its beneficial lipid changes. This may be due to bezafibrate acting as a pan-PPAR activator, as discussed below [15].

It has been argued that the lesser cardiovascular benefit observed in FIELD and BIP was due to inclusion of less dyslipidemic subjects than in the gemfibrozil trials. A post hoc subgroup analysis of BIP found a significant (40%) reduction in CHD in those with triglycerides ≥200 mg/dl [10]. VA-HIT found a similar trend toward increasing risk reduction with triglyceride levels ≥180 mg/dl [12]. In FIELD, fenofibrate, there were similar with reductions in cardiovascular risk in subjects with triglycerides less than and greater than 150 mg/dl. On the other hand, the Diabetes Atherosclerosis Intervention Study (DAIS) found that fenofibrate reduced angiographic progression of coronary atherosclerosis in a more markedly hypertriglyceridemic diabetic population [triglycerides 229 mg/dl (2.59 mmol/L); HDL 39 mg/dl (1.01 mmo/L); LDL 130 mg/dl (3.38 mmol/L)] [16]. However, when looking at the mean lipid levels across the studies, the case is less clear. The triglyceride levels in FIELD (172 mg/dl) were similar to those in the Helsinki Heart Study (178 mg/dl), but somewhat higher than in VA-HIT (160 mg/dl) and BIP (145 mg/dl) (Table 2). HDL levels were markedly lower in BIP (35 mg/dl) and in VA-HIT (32 mg/dl) than in either FIELD (43 mg/dl) or the Helsinki Heart Study (47 mg/dl). Taken as a whole, these findings may suggest that gemfibrozil may have a greater impact on cardiovascular risk than fenofibrate, regardless of the population studied.

Also of concern, some fibrates used alone may potentially increase the risk of cardiovascular and noncardiovascular mortality, and of serious adverse events (Table 1). Clofibrate, the earliest fibrate studied, is rarely used due to a consistent increase in mortality when compared to placebo, which occurred despite a substantial reduction in CHD events [17, 18]. In BIP, more cases of CHD mortality were reported for the bezafibrate group compared to placebo, although the difference was not statistically significant (Table 1) [10]. In FIELD, there were also more adverse events and deaths among those receiving fenofibrate compared to placebo [11]. The reduction in nonfatal coronary events and stroke in FIELD was counterbalanced by an 11% increase in cardiovascular deaths (due to a 19% increase in CHD death) and total mortality that did not reach statistical significance. The excess in deaths was due to a variety of causes: sudden cardiac death (70 versus 54, resp.), heart failure (13 versus 11), noncoronary cardiac (8 versus 4), and pulmonary embolism (4 versus 1, *P* = .22). Although a lower rate of cardiac events in the statin-treated placebo group is one possible explanation for the unexpected increase in cardiac deaths, a 30% excess of sudden death in the fenofibrate group is hard to explain if only an excess 9% of the placebo group received a statin. In contrast, fewer deaths occurred in the secondary-prevention population studied in the Veterans Affairs HDL Intervention Trial (VA-HIT) and in the primary-prevention Helsinki Heart Study [8, 9]. The secondary-prevention component of the Helsinki Heart Study reported a nonsignificant increase in CHD deaths with gemfibrozil compared to placebo in a much smaller sample (*N* = 628, HR 2.2% (95% CI 0.94–5.05)) [19]. It is important to note that no excess of harm has emerged in any statin trial. A meta-analysis of statin therapy in over 90,000 participants in 14 event trials found a 19% reduction in CHD mortality and a 12% reduction in all-cause mortality [20].

Although more malignancies were initially reported with clofibrate and gemfibrozil in 5-year primary-prevention trials, with long-term followup there were no significant increases in cancer incidence or mortality with gemfibrozil, even with followup as long as 18 years in the Helsinki Heart Study [8, 21, 22]. Cancer incidence was similar for both the fenofibrate and placebo groups (8%) in FIELD [11].

Also of concern in FIELD, cardiovascular events, including revascularizations, were significantly reduced only in those without previous cardiovascular disease and in those < 65 years of age (19% and 20%, resp.; *P* < .005), with no benefit (0%) observed in those with previous cardiovascular disease or who where ≥ age 65 years at baseline. These finding are in clear contradiction to the findings of the VA-HIT study where men with both diabetes and CHD experienced a 32% (95% CI 12–47, *P* = .004) reduction in cardiovascular events from gemfibrozil treatment [23]. The analysis has not been published to determine whether the explanation for the FIELD findings lies in the higher rate of crossover to other lipid-treatments in those with previous cardiovascular disease. In those with previous cardiovascular disease, 23% of the placebo group and 14% of the fenofibrate group crossed over to lipid-lowering therapy. In comparison, in those without previous cardiovascular disease, 16% of placebo and 7% of fenofibrate groups crossed over to statin therapy. On-treatment lipid values of the various groups were not reported so it is difficult to estimate whether the lack of benefit in those with previous cardiovascular disease and those ≥age 65 years was due to crossover to active treatment or to other factors.

In FIELD, the fenofibrate group also experienced a nonsignificant increase in deep venous thrombosis [67 (1.4%) versus 48 (1.0%); *P* = .74)]. No clear explanations for the nonsignificant higher rates of sudden death, venous thrombosis, and pulmonary embolism in FIELD are readily apparent. It is not known whether the increased risk of thrombosis was due to higher homocysteine levels in the fenofibrate group . Gemfibrozil may raise homocysteine levels less than fenofibrate [24]. It is not known whether the increased homocysteine levels resulted from the reversible increases in creatinine observed with fenofibrate, and also bezafibrate, and less commonly gemfibrozil [25]. Fenofibrate is known to raise homocysteine through a PPAR-*α* mediated mechanism [26]. Folic acid appears to lower fenofibrate-induced homocysteine elevations [27]. However, since clinical trials of folic acid supplementation to lower homocysteine have not demonstrated a reduction in cardiovascular events [28], the clinical importance of fenofibrate-induced homocysteine elevations remains to be established.

Nor is it clear that the increase in creatinine levels with fibrates increases cardiovascular risk since preliminary studies have shown that fenofibrate increases creatinine production rather than decreasing the glomerular filtration rate [25, 29]. In FIELD, progression of proteinuria and renal failure were less frequent in those receiving fenofibrate (Table 2) [11, 25]. No cases of renal failure were reported with gemfibrozil in the Helsinki Heart Study or in VA-HIT [8, 9].

All fibrates are known to increase biliary cholesterol saturation with clofibrate having the greatest effect and gemfibrozil the least effect [25]. In the World Health Organization (WHO) clofibrate primary prevention study, the excess mortality in the clofibrate group was due to a 33% increase in noncardiovascular mortality, including malignancy, postcholecyctomy complications, and pancreatitis [18]. Cholelithiasis and cholecystectomy rates were also higher in the Coronary Drug Project clofibrate arm and with gemfibrozil in the Helsinki Heart Study [17, 22]. In FIELD, although the rate of cholecystectomy was not reported, more cases of pancreatitis occurred in those receiving fenofibrate than placebo [40 (0.8%) versus 23 (0.5%), resp.; *P* = .31] [11].

Therefore, for a number of efficacy and safety reasons, fibrates should not be used indiscriminately for cardiovascular risk reduction. Furthermore, the role of fibrates for cardiovascular prevention is not clearly defined in the era of statin therapy. Statins are first-line therapy based on an extensive record of safety and efficacy in over 100,000 subjects to date, regardless of LDL or HDL level [30]. Whether adding a fibrate to statin therapy will reduce cardiovascular risk beyond that of statin monotherapy remains to be proven in the Action to Control Cardiovascular Risk in Diabetes (ACCORD) study to be completed in 2010 [31]. This trial will also evaluate the safety of adding fenofibrate to simvastatin therapy. In a corrected post hoc analysis of FIELD, when adjusting for the use of other lipid-lowering therapy, fenofibrate reduced major cardiovascular events by only 4% (95% CI −7 to 14, *P* = .45) [32]. It should be noted that this degree of risk reduction could simply be achieved by doubling the statin dose, which would lower LDL an additional 5–7% [33].

Safety is the other main concern with combination fibrate-statin therapy. There is consistent evidence that fibrates increase the risk of myopathy when used in combination with currently marketed statins. Fenofibrate is considered the fibrate of choice for those requiring statin therapy due to the lesser impact of fenofibrate on statin pharmacokinetics compared with gemfibrozil [25]. The risk of myopathy with gemfibrozil-statin therapy is about 30-fold higher than for fenofibrate-statin therapy [34]. When a gemfibrozil-statin combination is used in the highest-risk patients who are most likely to benefit (age ≥65 years with CHD and diabetes) the risk of rhabdomyolysis is almost 50-fold higher (1 in 484) than for statin monotherapy in unselected hospitalized patients [35].

Until more data become available, the addition of a fibrate to statin therapy should be reserved for patients at the highest near-term risk of cardiovascular death with elevated triglycerides and/or low HDL. In these patients, the reduction in deaths from cardiovascular causes by far outweighs any excess risk of death from noncardiovascular causes or of serious adverse events. This would include patients identified as very high risk by the U.S. National Cholesterol Education Program Adult Treatment Panel, such as those with cardiovascular disease with additional high risk characteristics, such as diabetes or metabolic syndrome, smokers, multiple risk factors, or those with diabetes and multiple poorly controlled risk factors, including smoking [30]. However, given the modest incremental benefit beyond that expected from its degree of LDL-lowering, the FIELD results may dampen enthusiasm for combination fenofibrate-statin therapy for the treatment dyslipidemia in the absence of severe hypertriglyceridemia (defined as ≥500 mg/dl [36]).

Even though gemfibrozil may be more effective for reducing cardiovascular events than fenofibrate, at least when used as monotherapy, concomitant use of gemfibrozil with a statin carries a much higher risk of myopathy than the fenofibrate statin combination . There were no cases of rhabdomyolysis in the 1000 subjects receiving both fenofibrate and statin therapy in FIELD [11]. Whether gemfibrozil is actually safer than fenofibrate would depend on the results of a head-to-head trial, although such a trial is unlikely to be performed. Marine omega-3 oils might prove to be a superior choice in terms of safety for the treatment of severe hypertriglyceridemia in patients requiring a statin therapy, especially in patients with impaired renal function since both fenofibrate and gemfibrozil have significant renal excretion [25]. Doses of omega-3 fatty acids of 3.4 grams or greater offer similar triglyceride-lowering efficacy to fibrates in some patient populations [37]. Although yet to be proven in a clinical trial in a population without high fish consumption, omega-3 fatty acids may also provide the added benefit of sudden death prevention and lower risk of total mortality [38].

Fibrates may also be reasonably considered for cardiovascular prevention in statin intolerant patients with dyslipidemia (for which gemfibrozil may be preferred). Fenofibrate has been shown to produce incremental improvements in triglycerides, HDL, and non-high-density lipoprotein (non-HDL) cholesterol used in combination with ezetimibe [39]. Fibrates are considered first-line drug therapy for the treatment of severe hypertriglyceridemia to prevent pancreatitis. Although clinical trials have not been performed to establish the morbidity and mortality benefits of treating severe hypertriglyceridemia, fibrates are very effective for treating triglyceride levels > 500 mg/dl [36]. It is not clear whether the small increase in pancreatitis risk with fenofibrate will increase the overall risk of pancreatitis in severely hypertriglyceridemic patients.

## 3. PPAR-*γ* AGONISTS: THIAZOLIDINEDIONES

Four large trials of TZDs with cardiovascular endpoints have now been reported. The first cardiovascular endpoint trial, the PROspective pioglitAzone Clinical Trial In macroVascular Events (PROACTIVE) study, enrolled over 5200 subjects with both diabetes and clinical CHD or peripheral arterial disease [40]. When acute coronary syndromes, revascularization, and amputation were included along with the accepted “hard” endpoints of nonfatal myocardial infarction, stroke, and total mortality in the primary endpoint, pioglitazone was not of significant benefit [HR 0.90% (95% CI 0.80 to 1.02), *P* = .095] (Table 3). However, for the secondary endpoint of nonfatal myocardial infarction, stroke, and total mortality, those receiving pioglitazone experienced a significant 16% reduction over the 3 years of the trial. The 16% reduction in ischemic events and death appears to be better than expected for the degree of lipid changes (Figure 1). The approximate 9% decrease in risk from the increase in HDL with pioglitazone might have been counterbalanced by the 2% increase in risk due to the 2% increase in LDL (Table 4) for a net expected cardiovascular risk reduction of 7%. Based on a meta-analysis, the 0.5% absolute decrease in hemoglobin A1c (HbA1c) would be expected to result in a 6-7% decrease in cardiovascular risk [41]. Thus, it appears that the reduction in cardiovascular risk observed with pioglitazone is similar to the expected 14% reduction from the combined changes in HDL, LDL, and HbA1C.

The US Food and Drug Administration recently required that a ``black box'' warning for congestive heart failure be placed on the labels of both currently available TZDs, pioglitazone and rosiglitazone [42]. TZDs, as a class, are well known to increase fluid retention through unknown mechanisms, which appear to be the primary contributor to the increased risk of congestive heart failure with TZDs [43, 44]. Fluid retention or edema occurs in 3–5% of patients with diabetes started on TZDs and upto 15% of patients treated with both TZDs and insulin [45, 46]. In PROactive, more cases of congestive heart failure occurred with pioglitazone (11%) compared to placebo (8%; *P* < .0001). The additional 56 cases of heart failure in the pioglitazone group directly counterbalanced the 55 fewer primary event endpoints (excluding silent myocardial infarctions). Despite 25 of the 47 cases of fatal heart failure occurring in the pioglitazone group, those receiving pioglitazone still had fewer deaths, 177 versus 186, although this was not statistically significant. In the Figure 1, when the increased risk of congestive heart failure is combined with the reduction in nonfatal MI, stroke, and death, pioglitazone performs worse than expected based on the lipid changes and appears to obviate the reduction in risk from improved glucose control. Taken together, these findings suggest that overall cardiovascular prevention is not a significant benefit of pioglitazone. There is a suggestion, however, that pioglitazone may have a net cardiovascular benefit over a period as short at 3 years if a method to prevent the fluid retention of TZDs is found.

On the other hand, rosiglitazone may not provide any clear cardiovascular benefits, and indeed there is concern that rosiglitazone may increase CHD risk. In a recent meta-analysis of 42 trials of at least 24 weeks duration, Nissen and Wolski found that those receiving rosiglitazone had a 43% higher risk of myocardial infarction and a 64% higher risk of cardiovascular death [47]. However, substantial methodologic limitations prevent definitive conclusions from being drawn regarding the safety of rosiglitazone from this analysis [48]. In the 3 large, long-term trials of rosiglitazone reported to date, findings have been mixed regarding its benefits [49–51]. Two trials were performed in subjects with type 2 diabetes, and 1 trial was for diabetes prevention. In all 3 trials, nonsignificant increases in nonfatal and fatal myocardial infarctions occurred in the rosiglitazone compared to control groups (Table 3). However, in all 3 trials, total mortality was lower in the rosiglitazone-treated groups, albeit again not achieving statistical significance. Since myocardial infarction, stroke, and death rates were low over the 3-4 years of observation in these trials, they were not powered to detect a difference in macrovascular events or mortality. As expected, all trials observed an increase in congestive heart failure, which further exacerbated the lack of cardiovascular benefit for rosiglitazone compared to control.

Both currently approved TZDs lower HbA1c by 1% when used alone or in combination in patients with poorly controlled diabetes [45, 46]. Both TZDs modify lipids to a lesser degree than fibrates. Rosiglitazone, however, appears to increase HDL half as much and LDL twice as much as pioglitazone [52]. The only TZD endpoint trial reporting both baseline and end-of-study laboratory values was the A Diabetes Outcome Progression Trial (ADOPT), comparing rosiglitazone to metformin or glyburide [Table 4] [49]. About 35% of subjects dropped out of the rosiglitazone and metformin groups during the trial, and over 45% dropped out of the glyburide group, limiting conclusions that can be drawn regarding the relative cardiovascular effects of these agents. Acknowledging this limitation, in Figure 1, rosiglitazone performed about as well in terms of a reduction in cardiovascular events, even if congestive heart failure events were included, as would be expected from the lipid changes when compared to metformin. It is perhaps surprising that rosiglitazone performed much worse than expected when compared to glyburide. An analysis of a large insurance database suggested that the risk of cardiovascular events with rosiglitazone was higher than with metformin, but lower than with sulfonylureas [53]. Another analysis of a large Veterans Health Administration database, however, suggested no differences in overall mortality for those receiving metformin, sulfonylureas, or TZDs [54].

Only baseline lipids were reported for the Rosiglitazone Evaluated for Cardiac Outcomes and Regulation of Glycaemia in Diabetes (RECORD) trial [51]. Extrapolating the relative degree of lipid changes observed in a head-to-head comparison of rosiglitazone to pioglitazone [52], it can be seen in Figure 1 that the cardiovascular event rates in RECORD was about what was expected from the extrapolated lipid changes (4.5% increase in HDL and 4% increase in LDL, or a 1% expected decrease in cardiovascular risk). Rosiglitazone has a net cardiovascular harm when congestive heart failure is added to myocardial infarctions and strokes (131 events versus 113 events, crude relative risk 1.16). Unfortunately, neither lipids nor HbA1c were reported for the Diabetes REduction Assessment with ramipril and rosiglitazone Medication (DREAM) trial, which evaluated the effect of rosiglitazone for the prevention of type 2 diabetes in 5269 adults at high risk on the basis of impaired fasting glucose and/or impaired glucose tolerance [50].

Taken as a whole, these findings may suggest that rosiglitazone has adverse effects on both heart failure and non-heart failure cardiovascular events that outweigh any beneficial changes in HbA1c. It is possible that a period of treatment longer than 3-4 years is needed to demonstrate a reduction in cardiovascular events, and ongoing trials of rosiglitazone will help to address this question, the Bypass Angioplasty Revascularization Investigation 2 Diabetes (BARI 2D) Trial, Veterans Affairs Diabetes Trial (VADT), and ACCORD [31, 55, 56]. However, it should be noted that pioglitazone already appears to perform better than expected from its lipid-modifying effects over a period of 3 years. Pioglitazone has been shown to reduce inflammation additive to that of simvastatin therapy, an effect that appears to be related to improvements in insulin resistance [57]. As for fibrates, it remains to be established whether adding pioglitazone to statin therapy will provide additional cardiovascular risk reduction. Some data regarding this question may emerge from ACCORD if pioglitazone replaces rosiglitazone as part of the diabetes management regimen [58].

Safety concerns in addition to congestive heart failure have emerged for TZDs. Both pioglitazone and rosiglitazone have an increased fracture risk [46, 59]. This may influence net benefits in women, and in older men, with long-term use. Cancer rates were reported only for PROactive among the longer-term TZD trials. Rates were similar in both treatment groups, with the exception of bladder cancer which was more frequent in the pioglitazone group [40]. Once bladder cancers occurring within the first year of the study were excluded from the analysis, 6 of the 9 cases were in the pioglitazone group and the imbalance was not felt to be related to pioglitazone treatment by the investigators. There have not yet been sufficient long-term follow-up studies to confirm if this finding is other than chance. Given the short duration of the study, this finding could eventually be of importance since rodents have shown an excess of bladder cancers with pioglitazone despite in vitro antineoplastic effects [45, 60].

In sum, PROACTIVE demonstrated that pioglitazone can be used without a net excess of serious adverse cardiovascular effects to manage hyperglycemia in a population of patients with diabetes and advanced cardiovascular disease. Piogltiazone may have benefits other than cardiovascular prevention, including its use in combination with other agents to control glucose and prevent microvascular events in properly selected patients. Piogltiazone should be used with caution in patients with New York Heart Association (NYHA) functional class 1 and 2 heart failure and are contraindicated in patients with class 3 or 4 heart failure. [43]. There were consistently fewer atherosclerotic CHD and stroke events in those who received pioglitazone who had history of either CHD or stroke at baseline and the risk of congestive heart failure with pioglitazone was similar in those with and without CHD and with and without stroke [61, 62].

However, in PROactive, in addition to hospitalized and unhospitalized heart failure, 1 out of 10 patients experienced discomfort and concern from fluid retention not requiring hospitalization [221 excess cases of edema without heart failure, number needed to treat (NNT) = 12]. These findings confirm that pioglitazone should remain second- or third-line therapy for the treatment of diabetes in patients [63]. Given the suggestion that rosiglitazone may carry an excess of cardiovascular events beyond the expected increase in congestive heart failure, until more data from long-term studies are available, rosiglitazone should be avoided and pioglitazone used preferentially for glucose management if indicated. Long-term event trials will be needed necessary to establish both efficacy and safety of any future PPAR-*γ* agonists, especially in light of the earlier withdrawal of troglitazone due to excess hepatic toxicity the emerged in postmarketing experience.

## 4. DUAL AGONISTS

The dual PPAR-*α*/*γ* agonists, or glitazars, developed to date display significantly higher PPAR-*γ* affinity than PPAR-*α* affinity, although their affinity for PPAR-*α* is higher than that of clinically used fibrates [64]. The dual PPAR-*α*/*γ* agonists have also been a disappointment in terms of cardiovascular prevention. Muraglitazar came the furthest along in development, and appears to have compounded the worst properties of the PPAR-*α* and PPAR-*γ* agonists used separately. In another review by Nissen et al. of Phase 2 and 3 trials ranging from 24 weeks to 2 years in duration, muraglitazar had a more than 2-fold incidence of CHD and stroke over placebo [65]. The adverse impact on cardiovascular risk occurred despite superior glucose-lowering and HDL-raising over pioglitazone [66]. Despite some suggestion that fenofibrate may attenuate fluid retention from rosiglitazone [67], fluid retention with muraglitazar occurred at a rate significantly higher than placebo. Development of tesaglitazar, another dual PPAR-*α*/*γ* agonist, was also terminated in Phase 3 development due to impairments of renal function [25, 68]. Bezafibrate is a pan-PPAR activator [15] and was associated with increased cardiovascular mortality in the Bezafibrate Infarction Prevention study, despite a large increase in HDL and improvements in LDL and triglyceride levels [10]. A number of other glitazars, including ragaglitazar, farglitazar, and imiglitazar, some with even more impressive effects on HDL and LDL than muraglitazar, have been terminated in late stage clinical trials due to safety concerns including carcinogenic effects, liver function test abnormalities, anemia, and decreased blood counts in part due to fatty infiltration of the bone marrow, in addition to fluid retention [64, 69].

## 5. PPAR AGONISTS AND CARDIOVASCULAR PREVENTION—WHAT NEXT?

In regard to pioglitazone, and perhaps other drugs activating PPAR-*γ*, if the mechanism underlying excess fluid retention can be addressed, the benefits should begin to outweigh adverse effects when used in high-risk populations. In the absence of such atherapeutic advance, a gene strongly predicting fluid overload with PPAR-*γ* and dual PPAR *α*/*γ* has been identified. If replicated in larger populations, this genetic polymorphism may identify which patients are least likely to experience fluid overload, which should result in a net cardiovascular benefit, at least for pioglitazone [70].

Research into other dual PPAR*α*/*γ* agonists with an improved safety margin is ongoing [64]. Selective modulation has been described for both PPAR-*α* [71] and PPAR-*γ* [72] and could explain the variation in biologic activity of various PPAR ligands within the same pharmacologic class. Since PPARs control numerous genes, beyond those influencing lipid and glucose metabolism, it is not surprising that the diverse origins adverse effects with PPAR agonists appear to be compound-specific, rather than a result of activation of more than one PPAR. The selective PPAR modulator (SPPARM) approach has been proposed as a method for developing ligands that differentially regulate genes specific for desirable biological effects but devoid of adverse effects. Several selective dual PPAR agonists in development do not appear to have adverse effects on fat accumulation and edema [64]. Metaglidasen is one such compound [73]. To further enhance safety, partial selective agonists appear to be more desirable than potent agonists. For example, potent PPAR-*α* activators may increase insulin resistance, induce cardiac hypertrophy, and reduce cardiac function [74]. Since gemfirbrozil appears to be of greater benefit for cardiovascular prevention while fenofibrate appears to be safer, a potentially fruitful avenue of investigation may be using the SPPARM approach to characterize the differential patterns of gene activation in various tissues for these 2 drugs.

The more recently discovered PPAR-*δ* has also been found to be a powerful regulator of fatty acid catabolism and energy homeostasis [6]. PPAR-*δ* agonism has been shown to prevent weight gain, dyslipidemia, and fatty liver in animals fed high-calorie diets [7]. A synthetic PPAR-*δ* agonist, GW501516, has been shown to modestly increase HDL-C levels and enhance serum fat clearance in an early human study [75]. Pan PPAR-*α*, *δ*, *γ* agonists have the potential to address multiple aspects of the metabolic syndrome with a single medication. One such pan-agonist, netoglitazone, has improved cell and tissue selectivity and is undergoing Phase II and III trials [73].

As our understanding of the effects modulating genetic expression in a variety of tissues continues to develop, safe and effective drugs to prevent the complications of obesity and diabetes should emerge. Clearly, all such drugs will need to undergo rigorous evaluation in long-term morbidity/mortality trials early in their development. Appropriate composite endpoints in these trials will be needed to evaluate the net benefits of PPAR activating drugs.

## Figures and Tables

**Table 1:** Selected morbidity and mortality outcomes in large, long-term fibrate trials. CHD = coronary heart disease, CVD = cardiovascular disease, MI = myocardial infarction, NR = not reported, ns = reported as “not significant,” RR = Crude relative risk calculated form reported number of events; hazard ratio was not reported.

Event rates								

Study treatment	Nonfatal MI	CHD mortality	Nonfatal MI or CHD death	Total stroke	Cancer	Total mortality	Hospitalized CHF	
**Helsinki Heart** [9]								
Mean F/U 5.0 years								
Primary prevention								
Dyslipidemia								
High LDL								

Placebo	3.5%	0.64%	4.1%	NR	1.3%	2.1%		
*N* = 2030								

Gemfibrozil	2.2%	0.53%	2.7%	NR	1.5%	2.2%		
*N* = 2051								

Hazard ratio (95% CI)	RR 0.63	RR 0.83	0.66	NR	RR 1.15	RR 1.05		
	*P* < .02	*p* = NR	*P* < .02		*p* = NR	*p* = NR		

**VA-HIT** [8]								
Mean F/U 5.1 years								
CHD								
HDL < 40 mg/dl								
LDL < 140 mg/dl								

Placebo	14.5%	9.3%	21.7%	6.0%	10.9%	17.4%	13.3%	
*N* = 1267								

Gemfibrozil	11.6%	7.4%	17.3%	4.6%	9.9%	15.7%	10.6%	
*N* = 1264								

Hazard ratio (95% CI)	0.77	0.78	0.78	0.75	RR 0.91	0.89	0.78	
	(0.62–0.96)	(0.59–1.02)	(0.65–0.93)	(0.53–1.06)		(0.73–1.08)	(0.62–0.98)	
	*P* < .02	*P* = .07	*P* = .006	*P* = .10		*P* = .23	*P* = .04	

**BIP [10]**						**Noncardiac death**		
Mean F/U 6.2 years								
CHD								
Dyslipidemia								

Placebo	11.2%	5.7%	15.0%	5.0%	5.9%	4.2%
*N* = 1542						

Bezafibrate *N* = 1548	9.7%	6.1%	13.6%	4.6%	5.5%	4.3%

Hazard ratio (95% CI)	0.87	RR 1.07	0.91	RR 0.92	RR 0.93	RR 1.02		
	*P* = .18	*P* = .61	*P* = .26	*P* = .66 ns		*P* = .87		

**FIELD** [11]							**Laser therapy**	**Albuminuria not progressing/regressing**
Mean F/U 5 years								
Type 2 diabetes								
Dyslipidemia								
Low LDL								

Placebo	4.2%	1.9%	6%	3.6%	8%	6.6%	5.2%	
*n* = 4900								

Fenofibrate	3.2%	2.2%	5%	3.2%	8%	7.3%	3.6%	
*N* = 4895								

	0.76	1.19	0.89	0.90		1.11	0.70	RR 1.15 *P* = .002
Hazard ratio (95% CI)	(0.62–0.94)	(0.90–1.57)	(0.75–1.05)	(0.73–1.12)	RR 1.0	(0.95–1.29)	(0.58–0.85)	
	*P* = .01	*P* = .22	*P* = .16	*P* = .36		*P* = .18	*P* = .0003	

**Table 2:** Selected laboratory data from fibrate endpoint trials.

Mean baseline level (mg/dL (mmol/L))		Percent difference between treatment groups		
**Helsinki Heart** [76]		Gemfibrozil versus placebo		
		1 year	3 years	5 years

Total cholesterol	269 (6.98)	−11%	−10%	−9%
LDL	189 (4.90)	−11%	−10%	−9%
HDL	47 (1.22)	11%	10%	7%
Triglycerides	178 (2.01)	−39%	−37%	−33%
Non-HDL	222 (5.76)	−15%	−14%	−13%

**VA-HIT** [12]			Gemfibrozil versus placebo	
			1 year	

Total cholesterol	175 (4.53)		−4%	
LDL	112 (2.90)		0%	
HDL	32 (0.83)		6%	
Triglycerides	160 (1.81)		−31%	

**BIP** [10]			Bezafibrate versus placebo	
			1 year	

Total cholesterol	212 (5.49)		−5%	
LDL	148 (3.83)		−7%	
HDL	34.6 (0.90)		18%	
Triglycerides	145 (1.64)		−21%	

**FIELD** [11]		Fenofibrate versus placebo		
		4 months		End-of-study

Total cholesterol	194 (5.04)	−11%		−7%
LDL	119 (3.07)	−12%		−6%
HDL	42.5 (1.10)	5%		1%
Triglycerides	172 (1.94)	−29%		−22%

**Table 3:** Selected morbidity and mortality outcomes in large, long-term trials of PPAR-*γ* agonists. CHD = coronary heart disease, CVD = cardiovascular disease, MI = myocardial infarction, NR = not reported.

Event rates							
**PROACTIVE** [40]	**Nonfatal MI**		**Stroke**	**Nonfatal MI/stroke/any death**	**Total mortality**	**Hospitalized CHF**	**Cancer**
Mean F/U 2.9 years							
Type 2 diabetes							

Placebo	5.5%		4.1%	13.6%	7.1%	4%	4%
*N* = 2633							
Pioglitazone	4.6%		3.3%	11.6%	6.8%	6%	4%
*N* = 2605							
Hazard ratio	0.83		0.81	0.84	0.96	RR* 1.5	RR* 1.0
(95% CI)	(0.65–1.06)		(0.61–1.07)	(0.72–0.98)	(0.78–1.18)	*P =* .007	
				*P* = .03			

**DREAM** [50]	**All MI**	**CVD death**	**Stroke**	**Nonfatal MI/stroke/CVD death**	**Total mortality**	**CHF**	**Diabetes**
MedianF/U 3.0 years							
Glucose intolerance							


Placebo	0.3%	0.4%	0.2%	0.9%	1.3%	0.1%	25%
*N* = 2634							
Rosiglitazone	0.6%	0.5%	0.3%	1.2%	1.1%	0.5%	10.6%
*N* = 2365							
Hazard ratio (95% CI)	1.66	1.20	1.39	1.39	0.91	7.03	0.38
	(0.73–3.80)	(0.52–2.77)	(0.44–4.40)		(0.55–1.49)	(1.60–30.9)	(0.33–0.44)
	*P* = .2	*P* = .7	*P* = .6	*P* = .2	*P* = .7	*P* = .01	*P* < .0001

**ADOPT** [49]	**All MI**		**Stroke**	**MI/stroke**		**CHF**	
Median F/U 4.0 years							
Type 2 diabetes							

Metformin (M)	1.5%		1.3%	2.8%		1.3%	
*N* = 1454							
38% drop-out rate							
Glyburide (G)	1.2%		1.2%	2.4%		0.6%	
*N* = 1441							
37% drop-out rate							
Rosiglitazone (R)	1.8%		1.1%	2.9%		1.5%	
*N* = 1456							
44% drop-out rate							
Hazard ratio (95% CI)	R versus M		R versus M	R versus M		R versus M 1.22 (0.66–2.26, *P =* .52) R versus G 2.20 (1.01–4.79, *P* = .05)	
	RR* 1.2		RR* 0.85	RR* 1.03			
	R versus G		R versus G	R versus G			
	RR* 1.5		RR* 0.92	RR* 1.21			

**RECORD** [51] interim	**All MI**	**CVD death**		**Nonfatal MI/stroke/CVD death**	**Total mortality**	**CHF**	
analysis							
Mean F/U 3.75 years							
Type 2 diabetes							

Metformin/sulfonylurea	1.8%	2.1%		5.1%	3.6%	1.0%	
*N* = 2227							
10% drop-out rate							
Rosiglitazone	2.2%	1.7%		4.9%	3.3%	2.1%	
added on to							
metformin/sulfonylurea							
*N* = 2220							
10% drop-out rate							
Hazard ratio (95% CI)	1.23	0.80		0.96	0.93	2.15	
	(0.81–1.86)	(0.52–1.24)		(0.74–1.24)	(0.67–1.27)	(1.30–3.57)	
	*P* = .34	*P* = .32		*P* = .74	*P* = .63	*P* = .003	

*RR = Crude relative risk; hazard ratio not reported.

**Table 4:** Selected laboratory data from endpoint trials of PPAR-*γ* agonists.

	Mean baseline level [mg/dL (mmol/L)]	Difference between treatment groups		
				End-of-study
**PROACTIVE** [40]				
HbA1c	7.9%		−6%	
LDL	112 (2.9)		2%	
HDL	42 (1.1)		9%	
Triglycerides	159 (1.8)		−13%	

**DREAM** [50]	HbA1c and lipids not reported			

**ADOPT** [49]	Median baseline level [mg/dL (mmol/L)]	Rosiglitazone versus Metformin		Rosiglitazone versus Glyburide

Glycated Hgb	7.4%	−2%		−6%
Total cholesterol	204 (5.28)	NR		NR
LDL	120 (3.11)	8%		5%
HDL	47 (1.22)	3%		6%
Triglycerides	161 (1.82)	−2%		−5%

**RECORD** [51, 77]	Mean baseline level [mg/dL (mmol/L)]			

Glycated Hgb	7.9%		NR	
LDL	127 (3.29)		NR	
HDL	46 (1.20)		NR	
Triglycerides	202 (2.28)		NR	
